# Quantitative Study of NPY-Expressing GABAergic Neurons and Axons in Rat Spinal Dorsal Horn*

**DOI:** 10.1002/cne.22570

**Published:** 2010-12-23

**Authors:** Erika Polgár, Thomas CP Sardella, Masahiko Watanabe, Andrew J Todd

**Affiliations:** 1 Institute of Neuroscience and Psychology, University of GlasgowGlasgow, G12 8QQ, UK; 2 Department of Anatomy, Hokkaido University School of MedicineSapporo 060-8638, Japan

**Keywords:** neurokinin 1 receptor, gephyrin, PKCγ, inhibitory interneuron, projection neuron, confocal microscopy

## Abstract

Between 25–40% of neurons in laminae I–III are GABAergic, and some of these express neuropeptide Y (NPY). We previously reported that NPY-immunoreactive axons form numerous synapses on lamina III projection neurons that possess the neurokinin 1 receptor (NK1r). The aims of this study were to determine the proportion of neurons and GABAergic boutons in this region that contain NPY, and to look for evidence that they selectively innervate different neuronal populations. We found that 4–6% of neurons in laminae I–III were NPY-immunoreactive and based on the proportions of neurons that are GABAergic, we estimate that NPY is expressed by 18% of inhibitory interneurons in laminae I–II and 9% of those in lamina III. GABAergic boutons were identified by the presence of the vesicular GABA transporter (VGAT) and NPY was found in 13–15% of VGAT-immunoreactive boutons in laminae I–II, and 5% of those in lamina III. For both the lamina III NK1r-immunoreactive projection neurons and protein kinase Cγ (PKCγ)-immunoreactive interneurons in lamina II, we found that around one-third of the VGAT boutons that contacted them were NPY-immunoreactive. However, based on differences in the sizes of these boutons and the strength of their NPY-immunoreactivity, we conclude that these originate from different populations of interneurons. Only 6% of VGAT boutons presynaptic to large lamina I projection neurons that lacked NK1rs contained NPY. These results show that NPY-containing neurons make up a considerable proportion of the inhibitory interneurons in laminae I–III, and that their axons preferentially target certain classes of dorsal horn neuron. J. Comp. Neurol. 519:1007–1023, 2011. © 2010 Wiley-Liss, Inc.

The spinal dorsal horn receives sensory inputs through central terminals of primary afferents and contains several populations of projection neurons, with axons that transmit information to the brain (Todd, 2010). Projection neurons are concentrated in lamina I (Rexed, 1952), largely absent from lamina II, and scattered through laminae III–VI. Many of those in laminae I, III, and IV express the neurokinin 1 receptor (NK1r) (Li et al., 1996, 1998; Marshall et al., 1996; Todd et al., 2000; Spike et al., 2003). Most dorsal horn neurons have axons that remain within the spinal cord and function as interneurons. These can be either excitatory (glutamatergic) or inhibitory, with the latter using gamma-aminobutyric acid (GABA) and/or glycine as their principal fast neurotransmitter(s) (Todd and McKenzie, 1989; Todd and Sullivan, 1990; Schneider and Lopez, 2002; Lu and Perl, 2003, 2005; Spike et al., 2003; Maxwell et al., 2007; Santos et al., 2007; Schneider and Walker, 2007).

The organization of neuronal circuits in the dorsal horn is still poorly understood, and a major reason for this is the difficulty in defining populations of interneurons. Immunocytochemical studies indicate that in laminae I–III inhibitory interneurons make up between 25 and 40% of the neuronal population, and that these are all GABAergic, with some using glycine as a cotransmitter (Todd and Sullivan, 1990; Polgár et al., 2003). The remaining neurons are glutamatergic (Todd et al., 2003), and these include projection cells and excitatory interneurons. Several attempts have been made to classify the interneurons based on morphological and/or electrophysiological criteria. For example, Grudt and Perl (2002) identified four major types of interneuron in lamina II: islet, central, vertical, and radial cells, and it has been shown that all islet cells are GABAergic (Todd and McKenzie, 1989; Todd et al., 1998; Lu and Perl, 2003; Maxwell et al., 2007; Yasaka et al., 2010). However, the relationship between morphology and transmitter content is less clear for the other three types (Hantman et al., 2004; Heinke et al., 2004; Lu and Perl, 2005; Maxwell et al., 2007), and a relatively high proportion of lamina II interneurons (≈25% in most studies) cannot be assigned to any of these classes (Grudt and Perl, 2002; Heinke et al., 2004; Yasaka et al., 2007, 2010).

An alternative strategy for classifying dorsal horn interneurons is based on their expression of neurochemical markers, including neuropeptides and various proteins (Todd, 2010). Certain neuropeptides, including neuropeptide Y (NPY), are restricted to GABAergic neurons in laminae I–III (Rowan et al., 1993), and most neurons in this region that contain the calcium-binding protein parvalbumin or the neuronal form of nitric oxide synthase (nNOS) are also GABA-immunoreactive (Antal et al., 1991; Spike et al., 1993). It has been shown that NPY, parvalbumin, and nNOS are not colocalized in laminae I–III (Laing et al., 1994), which suggests that these compounds may define distinctive populations of inhibitory interneurons. There is also evidence that the populations identified by these markers have specific roles in the neuronal circuitry of the dorsal horn. We have shown that GABAergic axons that contain NPY, and presumably originate from local interneurons, form numerous synapses on large NK1r-expressing projection neurons with cell bodies in lamina III and prominent dorsal dendrites, but form significantly fewer contacts on two other types of projection neuron: those in lamina I that express the NK1r and postsynaptic dorsal column cells in laminae III–V (Polgár et al., 1999b). In contrast, GABAergic axons that contain nNOS preferentially innervate a population of large lamina I projection neurons that lack the NK1r and have a very high density of inhibitory synapses that can be revealed with antibody against the glycine receptor-associated protein gephyrin (Puskár et al., 2001). Characterizing the inhibitory interneurons that are presynaptic to projection cells is important, as these may provide targets for the development of new analgesics. However, we do not know what proportion of GABAergic neurons or axonal boutons in laminae I–III contain NPY, the relative contribution made by these neurons to the inhibitory input to the lamina III NK1r projection cells, or whether all NPY-containing interneurons give rise to axons that innervate these cells.

We have therefore carried out a quantitative study in rat dorsal horn to address these issues. Since the majority of NPY-containing boutons in the superficial dorsal horn are not associated with the lamina III NK1r projection cells, we have also estimated the contribution that they make to the GABAergic innervation of two other types of neuron: the large gephyrin-coated projection cells in lamina I and interneurons in the inner part of lamina II (IIi) that express protein kinase Cγ (PKCγ; Mori et al., 1990; Malmberg et al., 1997; Polgár et al., 1999a).

## MATERIALS AND METHODS

### Animals

All experiments were approved by the Ethical Review Process Applications Panel of the University of Glasgow and were performed in accordance with the UK Animals (Scientific Procedures) Act 1986.

Tissue from 18 adult male Wistar rats (250–330 g; Harlan, Loughborough, UK) was used in this study. The animals were deeply anesthetized with pentobarbitone (300 mg intraperitoneally [i.p.]) and perfused through the heart with a fixative that contained 4% freshly depolymerized formaldehyde. Lumbar spinal cord segments (L2–L5) were removed, stored in fixative for 2–24 hours, then cut into 60-μm-thick sections in transverse, horizontal, or sagittal planes with a vibrating microtome.

For all of the immunocytochemical reactions described in subsequent sections, incubations in primary and secondary antibodies were carried out at 4°C. In all cases, apart from those that involved tyramide signal amplification (TSA), the antibodies were diluted in phosphate-buffered saline that contained 0.3M NaCl but no blocking serum. TSA reactions were performed according to the manufacturer's instructions.

### Proportion of neurons in laminae I–III that contain NPY

Transverse sections from the L4 segments of three rats were incubated for 3 days in a mixture of rabbit anti-NPY (Bachem, St Helens, UK; Cat. no. T-4070; 1:1,000) and monoclonal mouse antibody NeuN (Millipore, Watford, UK; Cat. no. MAB377; 1:1,000), and then overnight in species-specific secondary antibodies raised in donkey and conjugated to Alexa 488 (Invitrogen, Paisley, UK; 1:500) or Cy5 (Jackson Immunoresearch, West Grove, PA; 1:100). They were then incubated for 30 minutes at 37°C in propidium iodide (PI; Sigma-Aldrich, Poole, UK; 1%) in the presence of RNAse (Sigma-Aldrich; 10 mg/mL) to reveal cell nuclei (Todd et al., 1998).

Two sections from each rat were selected before NPY was viewed, and these were scanned with a Bio-Rad (Hercules, CA) Radiance 2100 confocal microscope, equipped with a blue diode (405 nm) argon multi-line, green HeNe (543 nm), and red diode (637 nm) lasers. Sections were scanned sequentially (to avoid fluorescent bleedthrough) with a 40× oil-immersion lens to produce z-series of 24 optical sections separated by 1 μm z-steps. Because of the limited area covered by the 40× lens, it was necessary to scan either 6 or 7 such z-series from each section to cover the entire cross-sectional area of laminae I–III.

**Figure 1:**
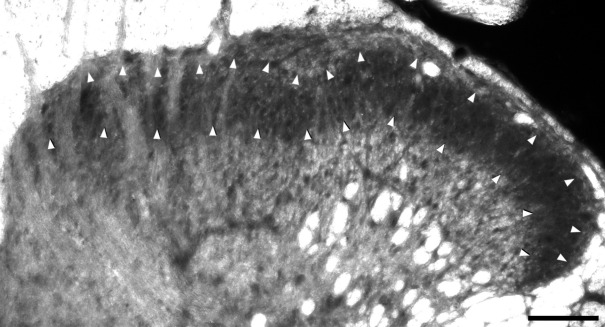
The dorsal horn seen with darkfield illumination. The two rows of arrowheads show the dorsal and ventral borders of lamina II, which appears as a dark band. Scale bar = 100 μm.

The sections were analyzed with a modification (Polgár et al., 2005) of the optical disector technique (Williams and Rakic, 1988; Coggeshall, 1992; Bjugn and Gundersen, 1993; Guillery, 2002). Merged images of NeuN and PI-staining were first viewed with Neurolucida for Confocal software (MicroBrightField, Colchester, VT). In each z-series the 14th optical section was designated as the reference section and the 22nd as the look-up section. Each optical section in the series was examined and the locations of all neuronal nuclei (defined by the presence of both NeuN-immunoreactivity and PI) that were present in the reference section or appeared in subsequent sections in the series were plotted onto an outline of the gray matter. All of those cells with nuclei that were still present on the look-up section were then excluded, leaving only neurons for which the bottom surface of the nucleus was located between reference and look-up sections. The depth at which the nucleus appeared largest was also noted for each cell included in the disector sample so that we could subsequently compare the depths of NPY-positive and NPY-negative neurons. The green channel (corresponding to NPY-immunoreactivity) was then viewed and the presence or absence of NPY-immunostaining in each of the selected neurons was determined. Boundaries between laminae I, II, and III were identified from low-magnification images obtained with light transmitted through a darkfield condenser (Todd et al., 1998), while the location of the ventral border of lamina III was determined from an atlas of rat spinal cord (Molander et al., 1984). With darkfield illumination, lamina II appears as a dark band due to the relatively low number of myelinated fibers and its border with lamina III can be readily identified (Fig. 1). Lamina I has a somewhat lighter appearance, due to the presence of more numerous myelinated axons. This lighter region can be identified in the middle and lateral parts of the dorsal horn, and we have found that it corresponds to the distribution of the dense plexus of NK1r-immunoreactive cell bodies and dendrites that occupies lamina I (Todd et al., 2008). In the medial third of the dorsal horn, the lamina I/II border is often less clear, but we have found, based on the distribution of NK1r-immunoreactivity (see above), that the width of lamina I in this region is ≈20 μm (E. Polgár and A.J. Todd, unpubl. obs.), and this was used to define the medial part of lamina I if its border with lamina II was not clearly seen with darkfield illumination.

Laminar borders were plotted on the drawings, and in this way we were able to determine the proportions of neurons in laminae I, II, and III that were NPY-immunoreactive. Since the number of lamina I neurons sampled in these sections was relatively small (19–28 cells per section), we also scanned lamina I from a further two sections from each of the three rats. These were selected and analyzed in the same way, except that only cells in lamina I were included.

Although the reference and look-up sections are relatively far apart, we ensured that all neurons with nuclei that lay between these two planes were included in the sample by examining every optical section between them. The positions of reference and look-up sections in the z-series were arranged so that perikaryal cytoplasm at both poles of the nucleus was visible in all cases. This was done to ensure that NPY immunoreactivity could be detected even in weakly labeled neurons. No correction was made for tissue shrinkage, since our aim was to determine the proportion of neurons that were NPY-immunoreactive, rather than the absolute number of cells in a volume of tissue.

The L4 segment was used for this analysis since it is the largest part of the lumbar enlargement, and several previous quantitative studies of neurochemically defined interneurons (Todd et al., 1998; Polgár et al., 2006) and of projection neurons (Spike et al., 2003; Polgár et al., 2008, 2010) have been carried out on this segment. However, in order to optimize the use of tissue obtained from each animal, any of the segments L2–L5 were used in the remaining parts of the study.

### Proportion of VGAT-immunoreactive boutons in laminae I–III that contain NPY

Transverse sections from the L3 segments of three rats were incubated for 3 days in a mixture of rabbit anti-NPY (1:1,000), monoclonal mouse antibody against the vesicular GABA transporter (VGAT; Synaptic Systems, Göttingen, Germany, Cat. no. 131 011; 1:1,000) and guinea pig anti-PKCγ (Yoshida et al., 2006; 1:500), and then overnight in secondary antibodies (all raised in donkey) conjugated to Alexa 488 (Invitrogen; 1:500), Rhodamine Red or Cy5 (both from Jackson Immunoresearch, 1:100).

Two sections from each of the three animals were selected and scanned sequentially with the confocal microscope through a 60× oil-immersion lens with a z-separation of 0.3 μm. For each section a set of three or four z-series (each containing at least 24 optical sections) was acquired in such a way as to cover a strip through the central part of laminae I–III. The choice of sections was based on the evenness of their upper surfaces, as this facilitated the scanning of a dorsoventral strip through these laminae.

The scans were analyzed with Neurolucida for Confocal. Initially, scans corresponding to VGAT immunostaining were viewed, and those from the same section were aligned so that the full thickness of laminae I, II, and III within the scanned strip was revealed. Laminar boundaries were identified as described above, except that in addition the ventral border of the dense plexus of PKCγ-immunoreactive dendrites that occupies the inner half of lamina II (Hughes et al., 2003) was used to confirm the lamina II/III border. These were drawn onto an overlay, and a 5 × 5 μm grid was placed over the confocal image stacks. A single optical section (the 12th in the z-series) was examined, and 100 VGAT-immunoreactive boutons that were present in this section were selected from each lamina. This was done by selecting the VGAT boutons located nearest the bottom right corners of the grid squares. For each lamina the first bouton was obtained from one of the most dorsal squares and the selection process then continued with squares in a dorsal-to-ventral, followed by left-to-right, direction until 100 boutons had been acquired. Once the selection was completed the files corresponding to NPY immunoreactivity were viewed and the presence or absence of NPY in each of the selected boutons was noted. Since this selection method will inevitably be biased towards boutons that were more extensive in the z-axis (Guillery, 2002), we compared the z-axis lengths of VGAT-immunoreactive boutons that contained NPY with those of VGAT^+^/NPY^−^ boutons. To do this, we chose the set of scans that contained the largest number of NPY-immunoreactive boutons among the selected VGAT sample (40 NPY boutons, of which 17 were in lamina I, 18 in lamina II, and 5 in lamina III). For each of these 40 boutons we also examined the nearest of the selected VGAT-immunoreactive boutons that lacked NPY. The z-axis lengths of these boutons were then measured by counting the number of optical sections (0.3 μm z-spacing) on which they appeared. This measurement was performed by an observer who was blind to the presence or absence of NPY in the selected boutons.

Preliminary observation suggested that all of the NPY-immunoreactive axonal boutons in laminae I–III were also VGAT-immunoreactive. To confirm this, we used Neurolucida for Confocal to select 100 NPY-positive boutons from one of the sets of confocal images for each rat. The selection was made before the images representing VGAT were viewed, and the selected boutons were distributed throughout the full thickness of laminae I–III. The VGAT image stack was then opened and the presence or absence of VGAT immunoreactivity was noted for each of the selected boutons.

### Analysis of GABAergic contacts on NK1r-immunoreactive lamina III projection neurons

Sagittal sections from three rats were incubated for 3 days in rabbit anti-NPY (1:500), guinea pig anti-NK1r (Polgár et al., 1999b; 1:1,000), and mouse anti-VGAT (1:1,000), and then overnight in appropriate donkey secondary antibodies conjugated to Alexa 488, Rhodamine Red, or Cy5. Sections were viewed with epifluorescence illumination and 15 of the large NK1r-immunoreactive neurons with long dorsal dendrites that entered lamina II were chosen (between four and six from each of the three rats). Selected regions of the dendritic trees of these cells were then scanned sequentially with the confocal microscope through a 60× oil-immersion lens and a z-separation of 0.5 μm. To avoid bias towards regions that were densely innervated by NPY-containing axons, the selection of cells and of the regions of dendritic tree to be scanned for analysis was made before the NPY immunoreactivity was viewed. For each cell, the length of dendrites analyzed for contacts was at least 200 μm.

Confocal images corresponding to NK1r and VGAT were viewed with Neurolucida for Confocal. The selected regions of dendrite were drawn and all contacts that they received from VGAT-immunoreactive boutons were plotted. The files corresponding to NPY were then viewed and the presence or absence of NPY in each of these VGAT boutons was noted. The locations of the borders between laminae I, II, and III were identified with a darkfield condenser and added to the drawing, and the proportion of VGAT boutons contacting the cells that were NPY-immunoreactive was determined for dendrites in each of these laminae.

### Analysis of inputs to lamina I gephyrin-coated neurons

Horizontal sections from three rats were incubated for 3 days in a cocktail consisting of sheep anti-NPY (Millipore, Cat. no. AB1583; 1:500), rabbit anti-VGAT (Synaptic Systems, Cat. no. 131 002; 1:1,000), and mouse monoclonal anti-gephyrin (Synaptic Systems, Cat. no. 147 011, clone 7a; 1:100,000). NPY and VGAT were detected with donkey secondary antibodies conjugated to Alexa 488 or Cy5, while gephyrin was revealed with a TSA kit (tetramethylrhodamine; PerkinElmer Life Sciences, Boston, MA).

Sections were viewed with epifluorescence illumination and 15 large gephyrin-coated lamina I neurons (Puskár et al., 2001; Polgár et al., 2008) were chosen (4–6 from each rat). Selected regions of dendritic trees, and in some cases also the soma, were scanned sequentially with the confocal microscope through a 60× oil-immersion lens, with a z-separation of 0.5 μm.

Confocal images corresponding to gephyrin and VGAT were initially opened in Neurolucida for Confocal and the selected regions of dendrite and soma were drawn. From each cell at least 100 of the VGAT-immunoreactive boutons that contacted gephyrin puncta were plotted onto these drawings. The files representing NPY-immunoreactivity were then opened and the presence or absence of NPY in each of the selected VGAT boutons was noted. Selection of the cells, the regions of dendrite to be analyzed, and the VGAT boutons contacting gephyrin puncta was made before NPY immunoreactivity was viewed.

### Analysis of GABAergic contacts on PKCγ-immunoreactive lamina II neurons

Sagittal sections from three rats were incubated for 3 days in rabbit anti-NPY (1:500), guinea pig anti-PKCγ (1:500), and mouse anti-VGAT (1:1000), and then overnight in appropriate donkey secondary antibodies conjugated to Alexa 488, Rhodamine Red, or Cy5. Between one and three sections were selected from each rat, and several adjacent fields from lamina IIi were scanned sequentially with the confocal microscope through a 60× oil immersion lens and a z-step of 0.5 μm. The files corresponding to PKCγ and VGAT were initially viewed with Neurolucida for Confocal and 15 PKCγ-immunoreactive neurons (five from each rat) were selected and drawn. Contacts that these cells received from VGAT-positive boutons on their cell bodies and dendrites were plotted onto the drawings and the presence or absence of NPY in each of these boutons was determined as described above. The selection of cells and plotting of VGAT contacts were performed before the NPY immunostaining was viewed. At least 100 μm length of dendrite was analyzed for each cell.

In order to confirm that NPY-immunoreactive boutons formed synapses on PKCγ cells, we incubated sagittal sections from two rats in a mixture of rabbit anti-NPY (1:1,000), guinea pig anti-PKCγ (1:500), and mouse anti-gephyrin (1:1,000) and detected these with donkey secondary antibodies conjugated to Alexa 488, Rhodamine Red, or Cy5. A single section from each rat was scanned through a 60× oil immersion lens at a z-step of 0.3 μm. Files corresponding to PKCγ and NPY were viewed with Neurolucida for Confocal and 100 contacts between NPY-containing boutons and cell bodies or dendrites of PKCγ-immunoreactive neurons were identified from each section. The gephyrin staining was then viewed and the presence or absence of a gephyrin-positive punctum in the cell membrane was determined for each contact.

### Comparison of NPY axons that innervate lamina III NK1r projection neurons with those that innervate the PKCγ-immunoreactive lamina II interneurons

In order to allow a direct comparison between NPY boutons that were presynaptic to the lamina III NK1r projection cells and those presynaptic to the PKCγ interneurons, sagittal sections from the L3 and L5 segments of two rats were incubated for 3 days in rabbit anti-NPY (1:1000), guinea pig anti-NK1r (1:1000), and mouse monoclonal anti-gephyrin (1:1,000) and then overnight in species-specific donkey antibodies against rabbit, guinea pig, and mouse IgGs that were conjugated to Rhodamine Red, Cy5, and Alexa 488, respectively. They were then incubated for 3 days in guinea pig anti-PKCγ (1:500), followed by biotinylated antiguinea pig IgG and avidin conjugated to Pacific Blue (Invitrogen; 1:1,000). Although both PKCγ and the NK1r were labeled with Pacific Blue in this tissue, the two neuronal populations could easily be distinguished in confocal scans, since the large NK1r-immunoreactive lamina III cells (which lack PKCγ; Polgár et al., 1999a) were also labeled with Cy5, while the PKCγ-immunoreactive interneurons were not. The NK1r was difficult to detect with fluorescence microscopy, since it was labeled with Cy5 (which is not readily visible) and only weakly with Pacific Blue. We therefore used the clusters of NPY-immunoreactive axons in laminae II and III to locate the large lamina III NK1r-expressing cells. These were then scanned sequentially through a 60× oil-immersion lens. Image stacks with a z-separation of 0.5 μm were scanned through parts of the dendritic trees of five NK1r cells from each rat, and in each case the scanned fields included nearby PKCγ-immunoreactive neurons in lamina IIi that received contacts from NPY-immunoreactive axons.

The z-series were examined with Neurolucida for Confocal. We identified NPY-immunoreactive boutons that were presynaptic to the NK1r projection cells or PKCγ interneurons based on the presence of gephyrin puncta in the somatic or dendritic membranes of these cells at the point of contact. All of these boutons were then marked on the Neurolucida overlay. Each of the NPY-immunoreactive boutons that was presynaptic to a PKCγ interneuron was then examined carefully to see whether an intervaricose axon that was connected to it could be followed in to the plexus of axons that innervated the lamina III NK1r cell.

To compare the size and immunofluorescence intensity of the two groups of NPY boutons (those presynaptic to the NK1r lamina III cells and those presynaptic to the lamina II PKCγ neurons), confocal images corresponding to NPY immunoreactivity were analyzed with MetaMorph software (Universal Imaging, Downington, PA). Care was taken to ensure that these images were scanned in such a way that pixels were not saturated. Each of the selected boutons was outlined with MetaMorph, and the maximum cross-sectional areas and average luminance values were recorded. In order to allow comparisons of pixel luminance values of NPY boutons from different scans, we normalized these values. This was done by measuring the mean of the luminance values for all of the NPY boutons that were presynaptic to the NK1r-immunoreactive cell on each confocal image stack and then determining the ratio of this value to that from the NK1r cell for which the mean value was highest. For each image stack the luminance values for all of the NPY boutons were then multiplied by the reciprocal of this ratio.

### Antibody characterization

Details of the primary antibodies that were used in this study are given in Table 1.

**Table 1:** Primary Antibodies

Antibody	Host	Antigen	Supplier/reference	Catalog number	Dilution
NPY	Rabbit polyclonal	Synthetic rat NPY	Bachem	IHC 7180 (T-4070)	1:500-1,000
NPY	Sheep polyclonal	Synthetic NPY conjugated to bovine thyroglobulin	Millipore	AB1583	1:500
NeuN	Mouse monoclonal	Purified cell nuclei from mouse brain	Millipore	MAB377	1:1,000
VGAT	Mouse monoclonal	Amino acids 75-87 of rat VGAT coupled to KLH	Synaptic Systems	131 011	1:1,000
VGAT	Rabbit polyclonal	Amino acids 75-87 of rat VGAT coupled to KLH	Synaptic Systems	131 002	1:1,000
NK1r	Guinea-pig polyclonal	Amino acids 393-407 of rat NK1r coupled to KLH	Polgár et al. (1999)		1:1,000
Gephyrin	Mouse monoclonal	Purified rat gephyrin	Synaptic Systems	147 011 (clone 7a)	1:100,000 (TSA) 1:1,000
PKCγ	Guinea pig polyclonal	Amino acids 684-697 of mouse PKCγ	Yoshida et al. (2006)		1:500

We have previously shown that immunostaining with the rabbit NPY antibody is abolished by preincubation with synthetic NPY (Rowan et al., 1993). It has been reported that immunostaining with the sheep antibody is absent in the brains of NPY knockout mice (Glavas et al., 2008). In addition, we found that dual immunofluorescence staining with the rabbit and sheep NPY antibodies revealed identical structures within the dorsal horn.

The mouse monoclonal antibody NeuN was generated against cell nuclei extracted from mouse brain and found to react with a protein specific to neurons (Mullen et al., 1992). We have shown that this antibody apparently labels all neurons (and no glial cells) within the rat spinal cord (Todd et al., 1998).

The two VGAT antibodies were raised against the same sequence, corresponding to amino acids 75–87 of the rat VGAT conjugated to keyhole limpet hemocyanin (KLH), and both antibodies stain bands of the appropriate molecular weight on western blots (Takamori et al., 2000; Guo et al., 2009). Immunofluorescence staining with both antibodies was blocked by preincubation with the immunizing peptide at 10^−6^M.

The guinea pig NK1r antibody was raised against a peptide corresponding to amino acids 393–407 at the C-terminus of the rat NK1r conjugated to KLH, and immunostaining with this antibody is abolished by preincubation with the immunizing peptide (Polgár et al., 1999b). We have also shown that it stains identical structures to those detected by a rabbit antibody (Polgár et al., 1999b), which gives no immunostaining in central nervous system (CNS) tissue from NK1r^−/−^ mice (Ptak et al., 2002).

The mouse monoclonal antibody against gephyrin was generated against an extract of rat spinal cord synaptic membranes (Pfeiffer et al., 1984) and has been extensively characterized and shown with western blots to bind to a 93-kDa peripheral membrane protein (gephyrin) in extracts of rat brain membranes (Becker et al., 1989; Kirsch and Betz, 1993).

The guinea pig PKCγ antibody, which was raised against a fusion protein of glutathione *S*-transferase (GST) and amino acids 684–697 of the mouse PKCγ, detects a single band of the appropriate molecular weight in western blots of brain homogenates from wildtype, but not PKCγ^−/−^, mice and immunostaining in the brain is also absent in the knockout mice (Yoshida et al., 2006).

Some sections were incubated in each of the fluorescent secondary antibodies that were used in the study, without having been incubated in primary antibodies. No staining was seen in these sections.

### Figures

Figures were composed with Adobe Photoshop (v. 8.0, San Jose, CA). In some cases image brightness and contrast were adjusted by using the levels setting.

### Statistical tests

The unpaired *t*-test, Mann–Whitney *U*-test, or Kruskal–Wallis analysis of variance (ANOVA) were used, as appropriate, and *P* < 0.05 was taken as significant.

## RESULTS

### General distribution of NPY in laminae I–III

**Figure 2:**
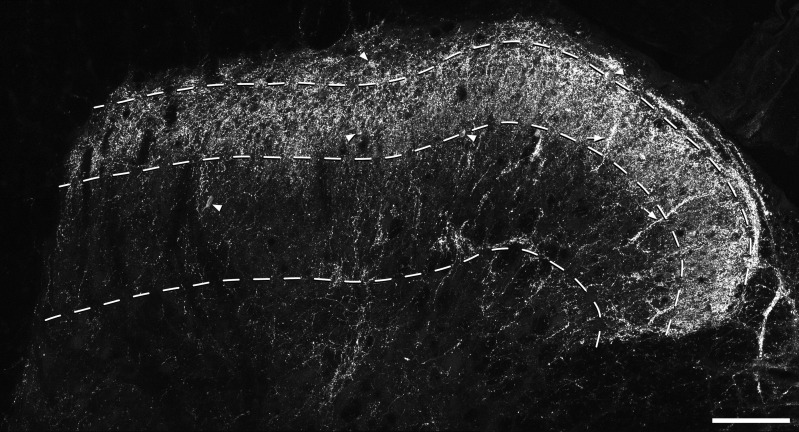
NPY-immunostaining in the dorsal horn. A confocal image from a transverse section through the dorsal horn immunostained to reveal NPY. The dashed lines represent the ventral borders of laminae I, II, and III. There is a dense plexus of immunoreactive axons that occupies laminae I and II, with some axonal staining in deeper laminae. Dorsoventrally orientated bundles of axons can often be seen and two of these are marked with arrows. Scattered immunoreactive cell bodies are present throughout laminae I–III and some of these are indicated with arrowheads. Note that many of those in laminae I–II are hidden by the axonal plexus. The image is a projection of 24 optical sections at 1-μm z-spacing. Scale bar = 100 μm.

In transverse sections stained for NPY, a dense plexus of immunoreactive axons occupied laminae I and II, and some axons were present in deeper parts of the dorsal horn (Fig. 2). Bundles of immunoreactive axons were often seen passing through the superficial laminae and penetrating into the deep dorsal horn, and many of these are likely to correspond to the clusters that have been shown to innervate the dorsal dendrites of lamina III NK1r-expressing projection neurons (Polgár et al., 1999b). At high magnification, the axonal immunostaining could be seen within boutons, and in some cases it was also present in the intervaricose portions that connected these boutons.

### NPY-immunoreactive cell bodies

**Figure 3:**
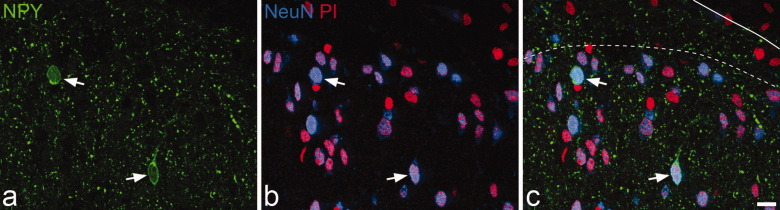
Confocal images showing NPY-immunoreactive neurons in laminae I–II. **a:** Part of a transverse section of the spinal cord immunostained to reveal NPY (green). **b:** The same field scanned for NeuN (blue) and the nuclear stain propidium iodide (red). In the merged image **(c)** the solid line indicates the edge of the gray matter, while the dashed line represents the lamina I/II border. Two NPY-immunoreactive cells (arrows) can be seen in lamina II. In each case the cell is NeuN-positive. Many other neurons that are not NPY-immunoreactive are visible. Nonneuronal nuclei lack NeuN, and therefore appear red. The small green profiles correspond to NPY-immunoreactive axons and their varicosities. The images were obtained from a single optical section. Scale bar = 10 μm.

**Figure 4:**
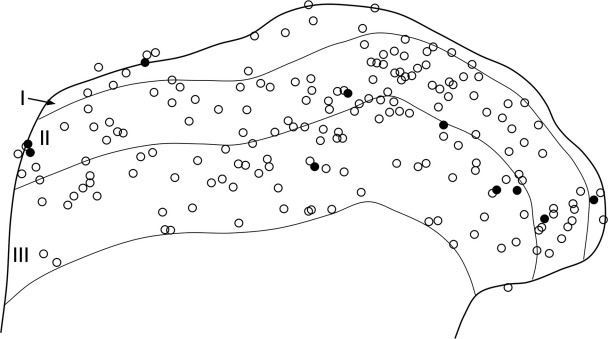
The distribution of NPY-immunoreactive and nonimmunoreactive neurons in laminae I–III. A plot of all of the neurons included in the disector sample from one of the sections that was used to determine the proportion of neurons that were NPY-immunoreactive. NPY-positive cells are shown as filled circles, while the remaining neurons are shown as open circles.

NPY-immunoreactive cells bodies were found throughout laminae I–III, and a few were also seen in deeper laminae. The NPY staining in these cells was located in the perikaryal cytoplasm and all were NeuN-positive, indicating that they are neurons (Fig. 3). Immunostaining with NPY antibody penetrated through the full thickness of the sections, and NPY-immunoreactive cell bodies were seen throughout the depth of the sections. Quantitative analysis with the disector method revealed that NPY-immunoreactive neurons constituted 5.8, 5.4, and 3.8%, respectively, of the total neuronal populations in laminae I, II, and III (Table 2; Fig. 4). We measured the mean depth in the z-series at which the nucleus of each of the neurons included in the disector sample appeared largest. For the NPY-negative cells (n = 1295) this value was 10.5 (±2.6 SD) μm below the top surface of the z-series, while for the NPY-positive cells (n = 68) it was 10.7 (±2.6 SD) μm below this surface. These values did not differ significantly (*P* = 0.5, *t*-test). This suggests that there was no reduction in the proportion of neurons that were NPY-immunoreactive at deeper levels within the z-series that were used for analysis.

**Table 2:** Percentages of Neurons in Laminae I-III That Were NPY-Immunoreactive

	Number of neurons counted	NPY-immunoreactive cells	% of neurons that were NPY- immunoreactive	% of neurons that are GABA- immunoreactive*	Estimated % of GABA neurons that contain NPY
I	97 (87 – 104)	5.7 (4 – 8)	5.8 (4 – 7.7)	24.8	23.4
II	192.3 (177 – 208)	10.7 (5 – 15)	5.4 (2.8 – 7.2)	31.3	17.3
III	165 (156 –177)	6.3 (4 – 8)	3.8 (2.5 – 4.5)	40.2	9.5

In each case the mean values for the three animals are shown, with the range in parentheses.

The percentages of neurons in each lamina that are GABA-immunoreactive are taken from the three naïve rats examined by Polgár et al. (2003).

### NPY in VGAT boutons

Quantitative analysis in the transverse sections that had been reacted for NPY, VGAT, and PKCγ revealed that NPY was present in 13% of VGAT boutons in lamina I, 14.7% of those in lamina II, and 5.3% of those in lamina III (Table 3). The mean z-axis lengths of NPY-positive and NPY-negative VGAT-immunoreactive boutons were 1.28 μm (range 0.9–2.1 μm, n = 40) and 1.31 μm (range 0.9–2.1 μm, n = 40), respectively. These values did not differ significantly (*P* = 0.67, *t*-test), indicating that a difference in size between the two types is unlikely to have distorted our estimate.

**Table 3:** Percentages of Boutons Among Different VGAT Populations That Were NPY-Immunoreactive

		VGAT boutons in contact with:		
		
Lamina	General population of VGAT boutons	Lamina III NK1r neurons	Lamina I gephyrin- coated neurons	Lamina IIi PKCγ neurons
I	13 (9-17) n = 600 (100)	32.4* (20-54.8) n = 521 (29-78)	6.3 (0-14) n = 2090 (100-238)	
II	14.7 (9-18) n = 600 (100)	35.6 (21.4-49.4) n = 1887 (77-199)	36.3 (21.2-51.9) n = 953 (48-94)	
III	5.3 (3-8) n = 600 (100)	29.6 (20.8-37.9) n = 1712 (29-302)		

Mean percentages of VGAT-positive boutons that were NPY-immunoreactive, with ranges shown in parentheses. The n values correspond to the total number of selected boutons analyzed, with the range of boutons selected for each section (for the general population) or cell (for contacts) in brackets. VGAT boutons in the general population were selected from six sections (two from each of three rats) with 100 boutons per lamina on each section. The boutons in contact with the different cell types were analyzed on 15 cells in each case.

* For the lamina III NK1r-immunoreactive neurons, contacts on dendrites in lamina I were analyzed on 12 of the 15 cells.

**Figure 5:**
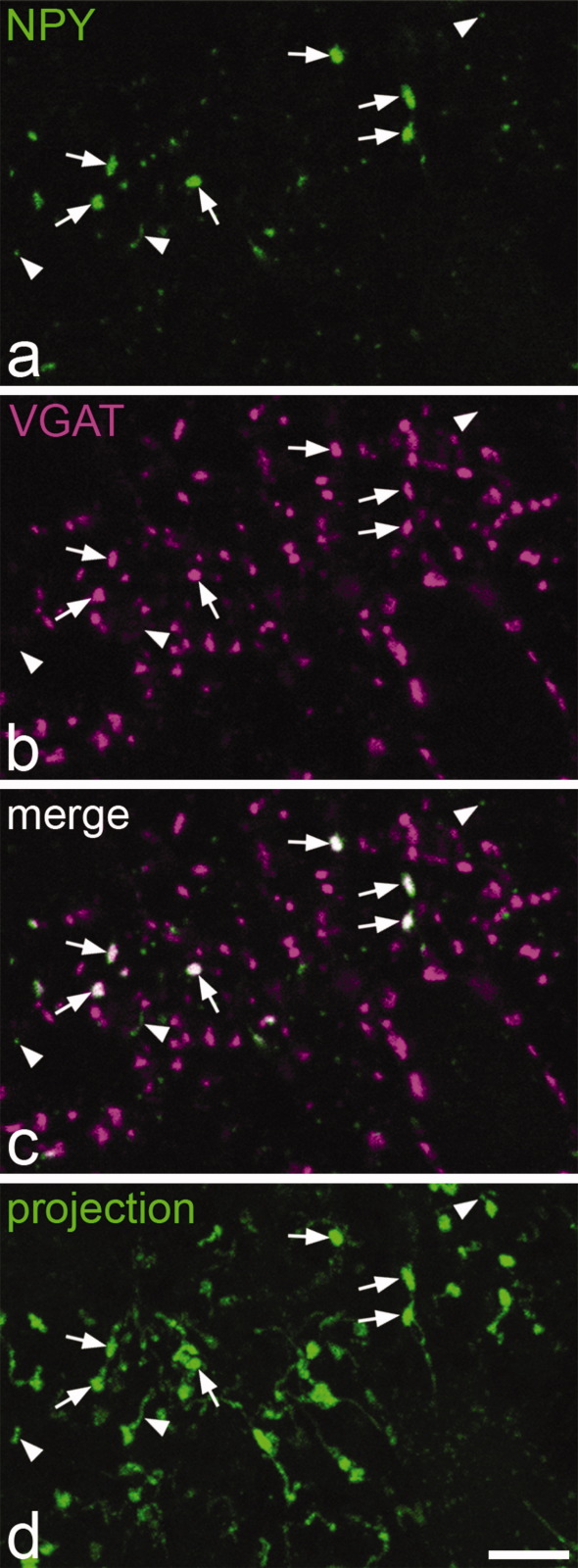
NPY and VGAT in the superficial dorsal horn. **a–c:** Confocal images of a single optical section showing part of lamina I in a transverse section that had been reacted to reveal NPY (green) and VGAT (magenta). Several NPY-immunoreactive axonal boutons are visible, and some of these are indicated with arrows. As seen in the merged image (c), all of these boutons are also VGAT-immunoreactive, but they are surrounded by many other VGAT-positive boutons that lack NPY. The very fine green profiles (three indicated with arrowheads) were found to be intervaricose portions of NPY-immunoreactive axons when followed through the series of optical sections, and these did not contain VGAT. **d:** A projection of 24 optical sections at 0.3 μm z-spacing from the same field shows the distinction between boutons (arrows) and intervaricose portions (arrowheads) of NPY-immunoreactive axons. Scale bar = 5 μm.

In the analysis of VGAT expression among NPY boutons, all of the selected NPY boutons (100 each from three rats) were VGAT-immunoreactive (Fig. 5), although VGAT was not detected in the intervaricose portions of NPY-containing axons.

### NPY boutons and lamina III NK1r-expressing cells

**Figure 6:**
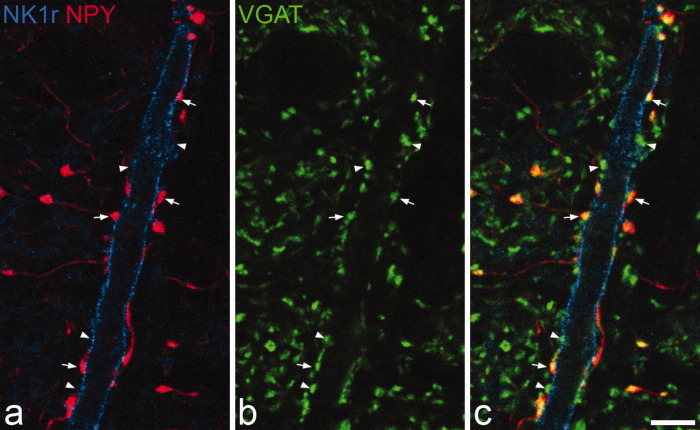
Contacts between NPY-immunoreactive boutons and a lamina III NK1r-expressing neuron. **a:** A confocal image from lamina II in a parasagittal section that shows NK1r-immunoreactivity (blue) on part of the dorsal dendrite of a lamina III neuron. The dendrite receives several contacts from boutons that are NPY-immunoreactive (red), some of which are indicated with arrows. **b:** The same field scanned to reveal VGAT (green). **c:** The merged image reveals that many VGAT-immunoreactive boutons that lack NPY are also in contact with the dendrite (some shown with arrowheads). The images are from a single optical section. Scale bar = 5 μm.

Contacts from VGAT-immunoreactive boutons on dendrites in laminae II and III were analyzed for all of the 15 lamina III NK1r-immunoreactive neurons. Contacts on dendrites of these cells that entered lamina I were examined in 12 cases, since for the remaining three cells their dorsal dendrites could not be followed into lamina I in the section that contained the cell body. Each of these neurons received numerous contacts from NPY-immunoreactive boutons; however, in all cases these were outnumbered by contacts from VGAT-immunoreactive boutons that lacked NPY (Fig. 6). For VGAT boutons that contacted these cells, the mean percentages that were NPY-immunoreactive were 32.4, 35.6, and 29.6 for laminae I, II, and III, respectively (Table 3). Kruskal–Wallis one-way ANOVA with all pairwise multiple comparisons (Dunn's method) revealed that for each lamina the proportions of boutons that contained NPY differed significantly between the general population of VGAT boutons and those that were in contact with the NK1r-immunoreactive lamina III neurons (*P* < 0.05).

### NPY boutons and lamina I gephyrin-coated cells

**Figure 7:**
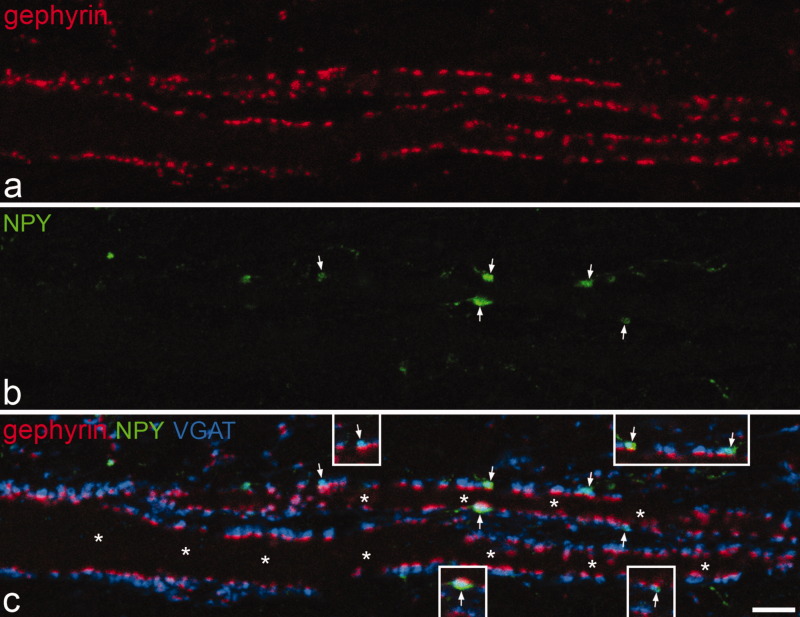
Contacts between NPY-containing boutons and a large gephyrin-coated lamina I neuron. **a:** Immunostaining for gephyrin (red, TSA method) in a horizontal section of lamina I shows parts of two proximal dendrites that belong to a large neuron with a cell body in lamina I. **b:** The same field scanned to reveal NPY (green). **c:** Merging the staining for gephyrin, NPY, and VGAT (blue) reveals that virtually every gephyrin punctum on the surface of the cell is associated with a VGAT-positive bouton, and that a few of these (marked with arrows) are NPY-immunoreactive. Asterisks in c indicate the two dendritic shafts. Note that the gephyrin puncta are relatively large (compared to those illustrated in Fig. 8) because they were detected with the TSA method. The images are projections of three optical sections at 0.5 μm z-spacing. The insets in c show single optical sections through each of the contacts from NPY-containing boutons. Scale bar = 5 μm.

All of the large gephyrin-coated lamina I neurons received a very high density of contacts from VGAT-immunoreactive boutons, with virtually every gephyrin punctum being associated with a VGAT bouton (Fig. 7). In all but one of these cases some of the VGAT boutons that contacted the cell were NPY-immunoreactive, with the mean proportion being 6.3% (Table 3). This differed significantly from the proportion of VGAT boutons in lamina I that were NPY-immunoreactive (mean 13%, see above; Mann–Whitney *U*-test, *P* < 0.005).

**Figure 8:**
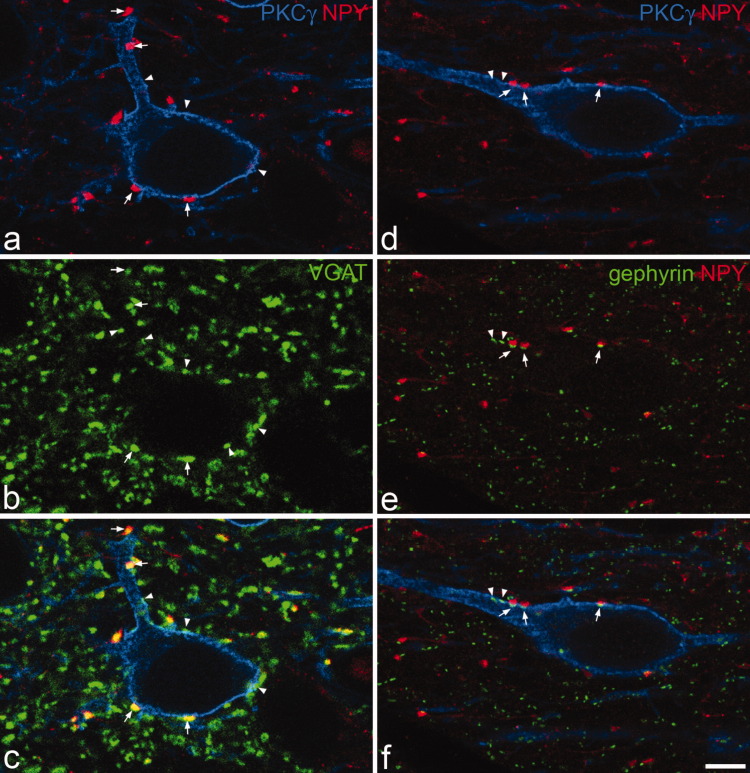
Contacts between NPY-containing boutons and PKCγ-immunoreactive interneurons in lamina IIi. **a–c:** show a field scanned to reveal PKCγ (blue), NPY (red), and VGAT (green) from a parasagittal section. A PKCγ-immunoreactive cell receives contacts on its cell body and dendrite from several VGAT-positive boutons, some of which also contain NPY. Arrows indicate some of the NPY boutons in contact with the cell and arrowheads show examples of VGAT boutons that do not contain NPY. **d–f:** A PKCγ cell (blue) in a section reacted to reveal NPY (red) and gephyrin (green). Arrows indicate three contacts onto the cell from NPY boutons and a gephyrin punctum is present in the cell membrane at each of these. Arrowheads show the locations of other gephyrin puncta that are in the cell membrane, but are not associated with NPY boutons. All images are from single optical sections. Scale bar = 5 μm.

### NPY boutons and PKCγ-expressing lamina II neurons

The 15 PKCγ neurons that were used to analyze contacts from VGAT/NPY boutons were located in lamina IIi, and had dendrites that remained in this region. All of the neurons received contacts from VGAT-containing boutons and in each case some of these were NPY-immunoreactive (Fig. 8a–c). Among the VGAT boutons that contacted the PKCγ neurons, 36.3% were NPY-immunoreactive (Table 3), and this differed significantly from the proportion that were NPY-immunoreactive among the general population of VGAT boutons in lamina II (mean 14.7%, see above; Mann–Whitney *U*-test, *P* < 0.001).

In the sections reacted to reveal NPY, PKCγ, and gephyrin we analyzed 100 contacts between NPY boutons and the cell bodies or dendrites of PKCγ-immunoreactive neurons in sections from each of the two rats (8 or 9 cells per animal, 7–19 contacts per cell). At virtually all (197/200, 98.5%) of these a gephyrin punctum was present in the cell membrane at the region of contact, and the cells also had many other gephyrin puncta in their membranes that were not associated with NPY boutons (Fig. 8d–f).

### Comparison of NPY boutons that formed synapses with NK1r or PKCγ cells

**Figure 9:**
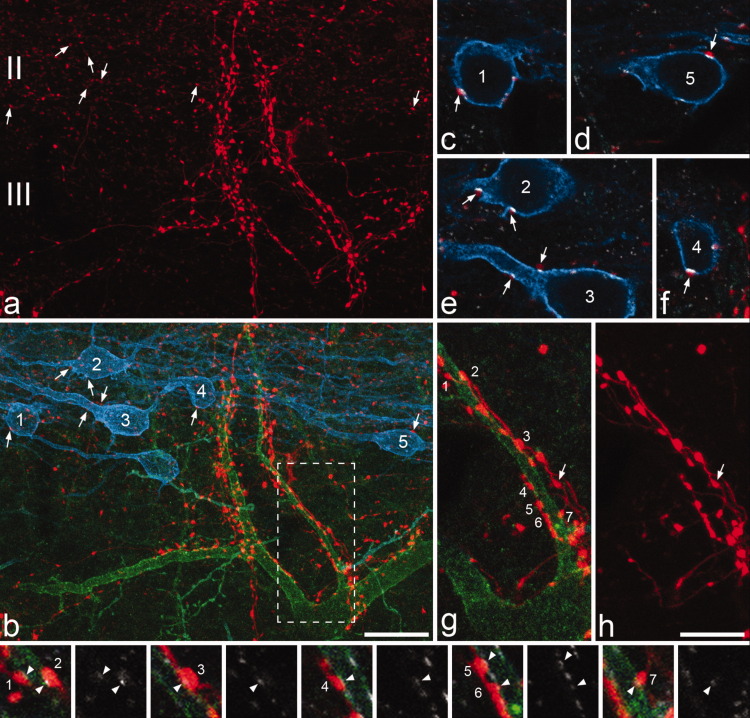
NPY axons that innervate NK1r lamina III cells and PKCγ lamina II interneurons appear to originate from different sources. **a,b:** Part of laminae II and III in a sagittal section that had been immunostained for NPY (red), NK1r (green), and PKCγ (blue). Several PKCγ-immunoreactive neurons can be seen and five of these are numbered. Each of these cells has contacts from NPY-immunoreactive boutons. Some of these are marked with arrows and shown at higher magnification in **c–f**. A large NK1r-immunoreactive cell is also present in this field and this has numerous contacts from NPY boutons. c–f: Single confocal optical sections scanned to reveal NPY (red), PKCγ (blue), and gephyrin (white) show that a gephyrin punctum is present at each of the contacts that the PKCγ cells receive from NPY boutons (arrows). **g,h:** Show contacts from NPY-containing boutons onto one of the dorsal dendrites of the NK1r cell at higher magnification and correspond to the boxed region in b. Seven of the NPY boutons that contact the cell are identified with numbers. The images in the bottom row, each of which is from a single optical section, are in pairs. In each case the left one was scanned for NPY (red), NK1r (green), and gephyrin (white), while the right one shows only gephyrin. Each of the contacts between the NPY boutons and the dendrite of the NK1r cell is associated with a gephyrin punctum, but these are much paler than those on the PKCγ cells. Note in h that the NPY-immunoreactive boutons that contact the NK1r cell are often linked by clear intervaricose portions, which indicates that they must originate from the same neuron. However, these axons also give rise to boutons that are not in contact with the NK1r cell (an example is indicated with an arrow in g,h). The NPY boutons that are presynaptic to the PKCγ cells are generally paler (as shown in a), and were never found to be connected by visible intervaricose portions to those that were presynaptic to the NK1r cells. a,b: Projections of 34 optical sections and g,h: of 8 optical sections at 0.5 μm z-spacing. Scale bars = 20 μm in a,b; 10 μm in c–h.

The scans that contained the 10 NK1r lamina III neurons also included 28 PKCγ-immunoreactive lamina II interneurons that received contacts from NPY-immunoreactive boutons (14 neurons in the sections obtained from each of the two rats). NPY boutons that formed contacts with the NK1r or PKCγ neurons were defined as being presynaptic to these cells if a gephyrin punctum was located in the somatic or dendritic plasma membrane of the cell at the point of contact (Fig. 9). Although we did not analyze this, we found that these gephyrin puncta in the membranes of the PKCγ neurons were generally much more intensely labeled than those in the membranes of the NK1r lamina III cells.

**Figure 10:**
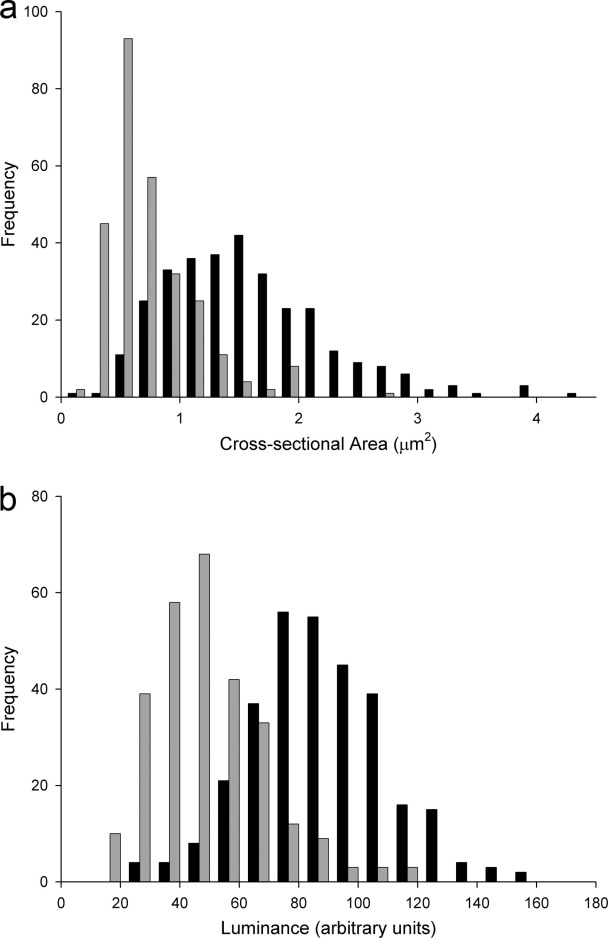
Differences between NPY boutons that were presynaptic to lamina III NK1r projection neurons and PKCγ interneurons in lamina IIi. Frequency histograms show (**a**) the cross-sectional area and (**b**) normalized mean luminance values for boutons that were presynaptic to the lamina III NK1r projection neurons (black bars, n = 309) or the PKCγ interneurons (gray bars, n = 280). In both cases the differences between these two populations were highly significant (*P* < 0.0001, Mann–Whitney *U*-test).

Altogether, 309 NPY boutons that were presynaptic to the NK1r cells (between 14 and 62 for each cell) and 280 NPY boutons that were presynaptic to the PKCγ cells (3–19 for each cell) were identified and analyzed. The NPY boutons that were presynaptic to the NK1r cells were generally larger and more brightly fluorescent than those that were presynaptic to the PKCγ cells (Figs. 9a,b, 10). The maximum cross-sectional areas for those presynaptic to the NK1r cells varied from 0.16–4.38 μm^2^ (median 1.44 μm^2^, n = 309), while the corresponding values for those presynaptic to the PKCγ cells ranged from 0.17–2.71 μm^2^ (median 0.61 μm^2^, n = 280), and this difference was highly significant (*P* < 0.0001, Mann–Whitney *U*-test). The mean luminance values for each NPY bouton were normalized to allow comparison of those from different scans (see Materials and Methods) and these are expressed in arbitrary units. Values for the NPY boutons presynaptic to the NK1r cells varied from 21–160 (median 84), while those for the boutons presynaptic to the PKCγ cells were between 16 and 114 (median 45). Again, the difference between these values was highly significant (*P* < 0.0001, Mann–Whitney *U*-test).

Most of the NPY-immunoreactive boutons that were presynaptic to the lamina III NK1r cells were attached to clearly visible intervaricose axons, and these could often be seen to connect several boutons that innervated the same NK1r cell (Fig. 9g,h). However, although intervaricose portions were sometimes seen on the NPY boutons that were presynaptic to PKCγ cells, these were never connected to boutons that were presynaptic to the nearby NK1r cell.

## DISCUSSION

The main findings of this study are: 1) that NPY-containing interneurons constitute 4–6% of the total neuronal population in laminae I–III, while NPY is present in 13–15% of GABAergic boutons in laminae I–II and 5% of those in lamina III; 2) that both lamina III NK1r projection neurons and lamina IIi PKCγ interneurons receive a relatively high proportion of their GABAergic input (approximately one-third) from boutons that contain NPY, but that there are significant differences in the size and immunostaining intensity between the populations of boutons that innervate the two cell types and these were not connected by intervaricose axons; and 3) that NPY-containing boutons are underrepresented among the GABAergic input to the large gephyrin-coated lamina I projection cells.

### Subpopulations of GABAergic interneurons

NPY-containing neurons in laminae I–III of the rat dorsal horn are all GABA-immunoreactive (Rowan et al., 1993) and based on results obtained with a physical disector method, we have estimated that GABAergic cells constitute 25%, 31%, and 40%, respectively, of the neurons in laminae I, II, and III in the L4–5 region of the rat dorsal horn (Polgár et al., 2003). Taken together with these previous estimates, the results of the present study suggest that the NPY-containing cells make up around 23% of the GABAergic neurons in lamina I, 17% of those in lamina II, and 9% of those in lamina III (Table 2). Since we have shown that lamina II contains ≈3.7 times as many neurons as lamina I in the L4 segment (Polgár et al., 2004), the proportion of GABAergic cells with NPY in laminae I and II together (the superficial dorsal horn) can be determined and is around 18%. It is possible that we have underestimated the proportion of neurons that contain NPY, since some cells may have contained the peptide at levels below the detection threshold. However, our results clearly indicate that a considerable proportion of the inhibitory interneurons in this region express NPY.

There is no evidence that NPY-containing axons project from the brain to the dorsal horn (Blessing et al., 1987), and it is therefore likely that the NPY-containing axons in laminae I–III originate from local inhibitory interneurons. This suggestion is supported by the finding that all of the NPY-immunoreactive boutons in these laminae were immunostained for VGAT. The proportions of VGAT boutons that contained NPY (13-15% in laminae I–II, 5% in lamina III) were somewhat lower than the estimated proportions of inhibitory interneurons that were NPY-immunoreactive (18% in laminae I–II, 9% in lamina III). This could be because the axons of the NPY neurons generate fewer boutons than those of other inhibitory interneurons, but may reflect the fact that this region also receives extrinsic GABAergic input, for example, from the rostral ventromedial medulla (Antal et al., 1996).

Two recent studies (Maxwell et al., 2007; Yasaka et al., 2010) have demonstrated that within lamina II those GABAergic neurons that are not islet cells are morphologically heterogeneous and include some central, vertical, and unclassified cells. It is not yet known whether these different groups correspond to discrete functional populations, and neurochemistry therefore provides a potentially powerful alternative method for classifying inhibitory interneurons in the superficial dorsal horn. We have previously demonstrated that NPY, nNOS, and parvalbumin are present in nonoverlapping populations of GABAergic neurons in laminae I–III (Laing et al., 1994). A major difference between the NPY-containing cells and those belonging to these other two populations is that while most nNOS- and parvalbumin-containing neurons are also glycine-immunoreactive (Spike et al., 1993; Laing et al., 1994), the NPY-containing neurons are not (Rowan et al., 1993). Since glycine receptors are widely expressed in the superficial part of the dorsal horn (Todd et al., 1996; Harvey et al., 2004), it is likely that fast transmission at synapses formed by the axons of the nNOS and parvalbumin interneurons (unlike those formed by axons of the NPY-containing cells) will involve a glycinergic component. Many glycinergic synapses in lamina II contain the GlyR α3 subunit, which is thought to be phosphorylated (and thus inhibited) by protein kinase A through a spinal action of prostaglandin E_2_ during peripheral inflammation (Harvey et al., 2004). Synapses formed by axons of the NOS- and parvalbumin-containing inhibitory interneurons (but not those formed by the NPY cells) are therefore likely to play a role in prostaglandin E_2_-mediated central sensitization in inflammatory hyperalgesia.

The NPY released by interneurons in laminae I–III will act on NPY Y1 and Y2 receptors on the central terminals of primary afferents, as well as on Y1 receptors that are expressed by many spinal cord neurons (Brumovsky et al., 2007). Presynaptic actions on primary afferents include a Y2-mediated reduction of substance P release (Moran et al., 2004), while the effects on dorsal horn neurons are inhibitory. Y1 receptors are expressed by several populations of neurons in the spinal cord, including many small interneurons located throughout laminae I–III, as well as by some projection neurons in lamina I (Brumovsky et al., 2006), and the role of NPY is therefore likely to be complex. Consistent with this, both antinociceptive and pronociceptive actions of the peptide have been reported (Brumovsky et al., 2007).

At present there is little information about the primary afferent inputs to the different neurochemical populations of inhibitory interneurons. However, we have recently found that following injection of capsaicin into the hindpaw ≈40% of NPY-containing cells, but very few nNOS- or parvalbumin-immunoreactive neurons, in the ipsilateral superficial dorsal horn contained phosphorylated extracellular signal regulated kinases (pERK) (S. Tiong, E. Polgár, and A.J. Todd, unpubl. data). Since pERK is a marker of neuronal activation (Ji et al., 1999), this suggests that many of the NPY neurons, unlike those that contain nNOS or parvalbumin, are activated by nociceptive primary afferents.

### Neuronal circuits involving NPY-containing interneurons

We have previously demonstrated that axonal boutons that contain NPY and GABA form synapses with the large NK1r-expressing lamina III cells (Polgár et al., 1999b), all of which are projection neurons (Todd et al., 2000). In the present study, we found that although cells of this type received numerous contacts from NPY/VGAT-immunoreactive boutons, these were outnumbered by approximately 2 to 1 by contacts from VGAT boutons that did not contain NPY. This indicates that these projection cells will also receive a substantial GABAergic input from other types of inhibitory interneuron and/or descending axons.

PKCγ-immunoreactive neurons are present throughout laminae I–III (Polgár et al., 1999a); however, they are clustered in the ventral half of lamina II, where their dendrites form a dense plexus (Hughes et al., 2003). During the course of this study we noticed that NPY-positive boutons frequently contacted PKCγ-immunoreactive cell bodies and dendrites, and subsequent quantitative analysis confirmed that over one-third of the VGAT boutons in contact with PKCγ cells in lamina IIi contained NPY. The presence of gephyrin puncta in the plasma membranes of these cells at virtually all of the sites of contact from NPY boutons indicates that these correspond to inhibitory (GABAergic) synapses.

In contrast, NPY-containing axons accounted for only ≈6% of the GABAergic boutons that formed synapses on the large gephyrin-coated lamina I projection cells. We have previously shown that around a quarter of the inhibitory input to these cells is derived from nNOS-containing interneurons (Puskár et al., 2001), and the low level of input from NPY-immunoreactive boutons seen in the present study provides further evidence that the inputs from inhibitory interneurons to projection cells are selectively organized.

The two populations that we have identified as major postsynaptic targets of NPY interneurons, NK1r-immunoreactive lamina III projection cells and PKCγ-expressing interneurons in lamina IIi, are likely to have very different functions. The lamina III projection cells are densely innervated by substance P-containing primary afferents (Naim et al., 1997), all of which are nociceptors (Lawson et al., 1997), and receive a modest input from low-threshold mechanoreceptive afferents (Naim et al., 1998). These cells have been shown to respond to noxious stimuli (Mantyh et al., 1995; Doyle and Hunt, 1999; Polgár et al., 2007), and their axons convey this information to various supraspinal targets, including the thalamus (Todd et al., 2000; Al-Khater et al., 2008). In contrast, the PKCγ neurons in lamina IIi are innervated by low-threshold myelinated afferents, but not nociceptors, and do not appear to respond to noxious stimuli (Neumann et al., 2008), although it has been suggested that they play a role in the development of neuropathic pain (Malmberg et al., 1997).

Our results strongly suggest that the NPY-immunoreactive boutons that innervate these two neuronal populations originate from different sets of inhibitory interneurons, since the boutons that formed synapses on the two cell types were never seen to be connected by intervaricose axons and differed significantly in both size and immunostaining intensity. The NPY interneurons that innervate the lamina III NK1r projection cells must also have other postsynaptic targets, since some of the boutons to which they were connected did not contact the projection cell. However, our finding that intervaricose axons could often be seen to connect several of the boutons presynaptic to a single NK1r projection neuron suggests that individual NPY interneurons can exert powerful inhibitory control over these cells, and therefore strongly suppress the transmission of nociceptive information to the brain.

If this interpretation is correct, then only a subset of NPY-containing interneurons will innervate the lamina III NK1r projection cells. Further studies involving whole-cell patch clamp recordings from NPY-expressing interneurons will be needed to determine whether these differ from those that innervate the PKCγ cells in terms of their laminar location, morphology, or physiological properties.
